# Skin autofluorescence is associated with plasma levels of matrix metalloproteinases in subjects without overt atherosclerosis

**DOI:** 10.1177/1358863X261420325

**Published:** 2026-03-31

**Authors:** Nynke A Jager, Helmy J Hinkema, Joop D Lefrandt, Damiano Baldassarre, Andries J Smit, Johanna Westra, Douwe J Mulder, Clark J Zeebregts

**Affiliations:** 1 Department of Rheumatology and Clinical Immunology, University Medical Center Groningen, University of Groningen, Groningen, The Netherlands; 2 Department of Internal Medicine, Division of Vascular Medicine, University Medical Center Groningen, University of Groningen, Groningen, The Netherlands; 3 Department of Pharmacological and Biomolecular Sciences, University of Milan, Milan, Italy; 4 Centro Cardiologico Monzino, IRCCS, Milan, Italy; 5 Department of Surgery, Division of Vascular Surgery, University Medical Center Groningen, University of Groningen, Groningen, The Netherlands

**Keywords:** advanced glycation end products, atherosclerosis, carotid artery disease, inflammation, matrix metalloproteinase, skin autofluorescence, vascular remodeling

## Abstract

**Background:**

Advanced glycation end products (AGEs) may promote arterial matrix remodeling via the receptor for AGEs (RAGE) signaling. Skin autofluorescence estimates tissue AGE burden. We examined whether skin autofluorescence relates to circulating matrix metalloproteinases (MMPs) and subclinical carotid atherosclerosis in asymptomatic adults.

**Methods:**

In a cross-sectional cohort of 262 asymptomatic adults, skin autofluorescence was measured with the AGE Reader. Plasma concentrations of MMP-3, MMP-9, tissue inhibitor of metalloproteinases-1 (TIMP-1), high-sensitivity C-reactive protein (hsCRP), and soluble RAGE (sRAGE) were measured by enzyme-linked immunosorbent assay (ELISA). Carotid intima–media thickness (IMT) and plaque burden were quantified by ultrasound. Associations between skin autofluorescence and biomarkers were tested using multivariable linear regression with prespecified covariates; biomarker coefficients were scaled per 10 ng/mL (hsCRP per 1 mg/L).

**Results:**

Skin autofluorescence correlated with MMP-3, MMP-9, TIMP-1, hsCRP, and IMT_mean_ (all *p* < 0.001) and with IMT_max_ (*p* = 0.008), but not with sRAGE. In multivariable models, skin autofluorescence remained independently associated with MMP-3 (*p* < 0.001), MMP-9 (*p* = 0.05), and TIMP-1 (*p* = 0.049), but not hsCRP (*p* = 0.41). Plaque-positive participants had higher skin autofluorescence, MMP-9, and hsCRP than those without plaques; MMP-9 and hsCRP were also higher in the high- versus intermediate-plaque group, whereas MMP-3 and TIMP-1 did not differ.

**Conclusions:**

In asymptomatic adults, higher skin autofluorescence is independently associated with circulating MMP-3, MMP-9, and TIMP-1, and with early carotid atherosclerosis markers. Findings support a link between tissue glycation and arterial matrix remodeling and suggest that skin autofluorescence plus MMP-9 may offer a practical, noninvasive approach for early vascular risk stratification.

## Background

Atherosclerosis is a complex and progressive vascular condition characterized by lipid accumulation, inflammation, and structural remodeling of the arterial wall.^1^ Advanced glycation end products (AGEs), which accumulate on long-lived proteins due to nonenzymatic glycation and oxidative stress, have been implicated in the development of vascular damage, particularly in diabetes mellitus (DM) and chronic kidney disease.^2,3^ These long-lived proteins include structural extracellular matrix components such as collagen and elastin, which have low turnover rates and are therefore particularly susceptible to AGE accumulation. Glycation of these proteins contributes to increased matrix stiffness and impaired vascular elasticity.^2^ AGEs may promote plaque formation and instability through their interaction with the receptor for AGEs (RAGE), triggering pro-inflammatory and matrix-degrading pathways.^4^

RAGE is expressed on several vascular and inflammatory cell types, including endothelial cells, vascular smooth muscle cells, and macrophages. Under physiological conditions, RAGE expression is low, but it is markedly upregulated in pathological settings such as DM, chronic inflammation, and atherosclerosis. Engagement of RAGE activates downstream signaling pathways, including nuclear factor-κB (NF-κB) and mitogen-activated protein kinases, thereby promoting sustained inflammatory signaling and extracellular matrix degradation.^2,4^ Matrix metalloproteinases (MMPs), a family of zinc-dependent endopeptidases, play a crucial role in extracellular matrix turnover and have been linked to all stages of atherogenesis, including plaque vulnerability.^5,6^ Clinical studies measuring AGEs in plasma are often limited by technical complexity and variability, whereas skin autofluorescence offers a reproducible, noninvasive alternative. Prior studies have shown elevated skin autofluorescence in patients with coronary artery disease, stroke, and peripheral artery disease, even in the absence of DM.^7–9^ This suggests that skin autofluorescence may reflect vascular risk beyond traditional factors, but its relation to early matrix degradation and vascular remodeling remains unclear. Experimental evidence suggests that AGEs stimulate MMP expression, but clinical validation in asymptomatic individuals remains limited.^10–12^ Noninvasive assessment of tissue AGEs using skin autofluorescence provides a practical tool for exploring this relationship in vivo.^13^

In this context, MMP-3 and MMP-9 were selected because of their complementary roles in atherosclerotic remodeling. MMP-3 contributes to early extracellular matrix turnover and activates other matrix metalloproteinases, whereas MMP-9 is strongly associated with vascular inflammation and plaque progression.^5,6^ Improved identification of early vascular remodeling may have important translational implications by enabling earlier risk stratification and preventive intervention before the development of clinically overt atherosclerotic disease.

This study aimed to investigate the relationship between tissue AGE accumulation, measured by skin autofluorescence, and circulating levels of matrix metalloproteinases (MMP-3 and MMP-9), the tissue inhibitor of metalloproteinases-1 (TIMP-1), systemic inflammation, and markers of subclinical atherosclerosis in a well-characterized cohort of asymptomatic individuals. We hypothesized that increased skin autofluorescence is associated with elevated plasma MMPs and carotid intima–media thickness (IMT), reflecting early vascular remodeling.

## Methods

### Study population

This cross-sectional study included 262 asymptomatic adults (⩾ 18 years) without a history of cardiovascular disease (CVD). Participants were recruited from two cohorts: (1) a low-risk group, consisting of volunteers aged 19–81 years with no more than one cardiovascular risk factor (RF), recruited via public advertisement, and (2) a higher-risk group derived from the IMPROVE study, a European cohort of individuals aged 55–79 years with at least three RFs.^14^ Analyses were performed in the combined cohort to preserve statistical power and reflect a continuum of cardiovascular risk. Individuals with established CVD, renal dysfunction (estimated glomerular filtration rate < 60 mL/min/1.73 m^2^), autoimmune disease, active cancer, or recent infections were excluded. All participants were White. Individuals with Fitzpatrick skin types V–VI were excluded due to limited reliability of skin autofluorescence measurement at low skin reflectance values.^15^ All participants provided written informed consent. The study protocol was approved by the local ethics committee of our tertiary referral center and was conducted in accordance with the STROBE guidelines for observational studies.^16^

### Cardiovascular risk factor definitions

RFs were defined according to standard clinical criteria.^17^ Hypertension was defined as systolic blood pressure ⩾ 140 mmHg, diastolic pressure ⩾ 90 mmHg, or use of antihypertensive medication. Dyslipidemia was defined as low-density lipoprotein (LDL) cholesterol > 4.0 mmol/L, high-density lipoprotein (HDL) cholesterol < 1.2 mmol/L (women) or < 1.0 mmol/L (men), triglycerides > 4.0 mmol/L, or current use of lipid-lowering therapy. Obesity was defined as a body mass index (BMI) ⩾ 30 kg/m^2^. DM was defined as known diabetes, a fasting plasma glucose ⩾ 7.0 mmol/L, or random plasma glucose > 11.1 mmol/L. Smoking status was categorized as current, former, or never. The 10-year cardiovascular risk was calculated using the Framingham Risk Score (FRS) algorithm, based on age, sex, cholesterol levels, smoking, and blood pressure.

### Measurement of skin autofluorescence

Skin autofluorescence was measured using the AGE Reader (DiagnOptics Technologies BV, Groningen, The Netherlands), a noninvasive desktop device that uses ultraviolet light (300–420 nm) to excite fluorescent AGEs in a 4 cm^2^ area of skin on the volar forearm. The volar forearm was selected as a standardized measurement site because it is relatively protected from ultraviolet exposure, and measurements were performed under standardized conditions to minimize the influence of sun exposure or topical skin products. Emission light (420–600 nm) is captured, and autofluorescence is calculated as the ratio of emitted to reflected light, expressed in arbitrary units (AU).^18^ Three consecutive measurements were averaged per subject. Measurements were performed at the same anatomical site on the volar forearm, with minimal repositioning of the device between measurements. Measurements were accepted only when skin reflectance exceeded 6%, ensuring validity in individuals with Fitzpatrick skin types I–IV.^18^ The method is operator-independent and validated against AGE accumulation in dermal biopsies.^13,19^

### Blood collection and biochemical analyses

Venous blood samples were collected after overnight fasting. Lipid profiles, glucose, and creatinine were analyzed using standard laboratory techniques. Plasma was stored at −80°C until batch analysis. High-sensitivity C-reactive protein (hsCRP) was measured by nephelometry (BNII N; Dade Behring, Germany). Plasma levels of MMP-3, MMP-9, TIMP-1, and sRAGE were determined by enzyme-linked immunosorbent assay (ELISA), following the manufacturers’ protocols.^20–22^ Intra- and inter-assay coefficients of variation were < 10%.

### Carotid ultrasound and plaque scoring

Carotid IMT was assessed by high-resolution B-mode ultrasound in the common, bifurcation, and internal carotid arteries in multiple projections, as described in the IMPROVE protocol.^14^ Both study groups were evaluated using similar ultrasound systems and far-wall IMT measurements. IMT_mean_ was calculated as the average of all measurements; IMT_max_ was defined as the highest observed value. Plaques were defined according to the Mannheim consensus: focal thickening of ⩾ 0.5 mm, > 50% thicker than surrounding IMT, or absolute thickness ⩾ 1.5 mm.^23^ A plaque score (0–6) was computed by summing affected segments. For descriptive and comparative analyses, participants were categorized into three groups based on plaque score: no plaques (score 0), intermediate plaque burden (one to three segments), and high plaque burden (four or more segments). These cut points were chosen to reflect increasing extent of atherosclerosis and are consistent with prior applications of the Mannheim consensus and the IMPROVE study protocol.^14,23^ Scans were performed by different trained sonographers across the two cohorts. Although inter-operator variability was not formally tested, all sonographers followed a standardized protocol, and image analysis was performed centrally by a blinded reviewer. Ultrasound analysts were blinded to clinical and laboratory data.

### Statistical analysis

Continuous variables were summarized as means ± SD or medians with IQR, depending on distribution (Kolmogorov–Smirnov test). Categorical variables were reported as frequencies and percentages. Between-group comparisons used ANOVA or Kruskal–Wallis tests (continuous data) and chi-squared tests (categorical data). Pearson or Spearman coefficients were used to assess correlations. Multivariable linear regression models were constructed using stepwise selection (entry criterion *p* < 0.1), with skin autofluorescence as the dependent variable. Independent variables included MMP-3, MMP-9, TIMP-1, hsCRP, sRAGE, and covariates (age, BMI, smoking, DM). IMT and plaque burden were not included as independent variables, as they were analyzed separately as outcome measures of subclinical atherosclerosis. Additional cardiovascular risk factors, such as hypertension and dyslipidemia, were not entered separately to avoid collinearity with age and medication use. Regression coefficients were scaled to clinically meaningful units (per 10 ng/mL for MMPs and per 1 mg/L for hsCRP) to facilitate interpretation. Medication use was not included in the multivariable models to avoid collinearity with the cardiovascular risk factors for which these treatments were prescribed. A two-tailed *p* < 0.05 was considered statistically significant. Analyses were performed in SPSS version 22.0 (IBM Corp., Armonk, NY, USA). Post hoc correction for multiple comparisons was not applied, as between-group analyses were exploratory and limited to predefined comparisons.

## Results

### Study population

A total of 262 individuals (131 men and 131 women) with a median age of 60.2 years (range: 19–81.5) were included. Baseline characteristics are presented in Table 1. The prevalence of traditional cardiovascular risk factors was high: 69% of participants had hypertension, 65% hyperlipidemia, 32% were obese, 12% had DM, and 15% were current smokers.

**Table 1.** Baseline characteristics and risk factors for atherosclerosis.

	Subjects (*N* = 262)
Male sex, *n* (%)	131 (50)
Age, years	60.2 (19–81.5)
BMI, kg/m^2^	27.8 ± 5.2
Obesity, *n* (%)	75 (29)
Smoking status, *n* (%)	
Current	41 (15)
Past	163 (61)
Never	58 (22)
Hypertension, *n* (%)	185 (69)
Systolic blood pressure, mmHg	146 ± 20
Diastolic blood pressure, mmHg	83 ± 10
Hyperlipidemia, *n* (%)	175 (65)
Triglycerides (mmol/L)	1.3 (0.5–5.6)
Total cholesterol (mmol/L)	5.3 ± 1.1
HDL (mmol/L)	1.3 ± 0.4
LDL (mmol/L)	3.3 ± 1.0
Diabetes mellitus, *n* (%)	33 (12)
Plasma glucose (mmol/L)	4.8 (2.7–13.8)
Medication, *n* (%)	
Statin use	74 (28)
ACEi or ARB use	52 (19)
Other antihypertensive medication	150 (56)
Aspirin use	14 (5)
Oral antidiabetic medication	21 (8)
Insulin use	5 (2)

Data are expressed as mean ± SD or median (range) unless otherwise noted.

ACEi, angiotensin-converting enzyme inhibitor; ARB, angiotensin receptor blocker; BMI, body mass index; HDL, high-density lipoprotein; LDL, low-density lipoprotein.

In univariable analyses, skin autofluorescence showed significant positive correlations with age (*r* = 0.36, *p* < 0.0001), BMI (*r* = 0.35, *p* < 0.0001), plasma glucose (*r* = 0.26, *p* < 0.0001), and the presence of diabetes (*p* < 0.001) and obesity (*p* = 0.0004). A negative correlation was observed with HDL cholesterol (*r* = −0.23, *p* = 0.0002). No significant correlations were found with LDL cholesterol, total cholesterol, triglycerides, or blood pressure. Skin autofluorescence did not differ between sexes. Similarly, there were no sex differences in MMP levels or plaque burden.

### Skin autofluorescence and circulating biomarkers

**Figure 1.**
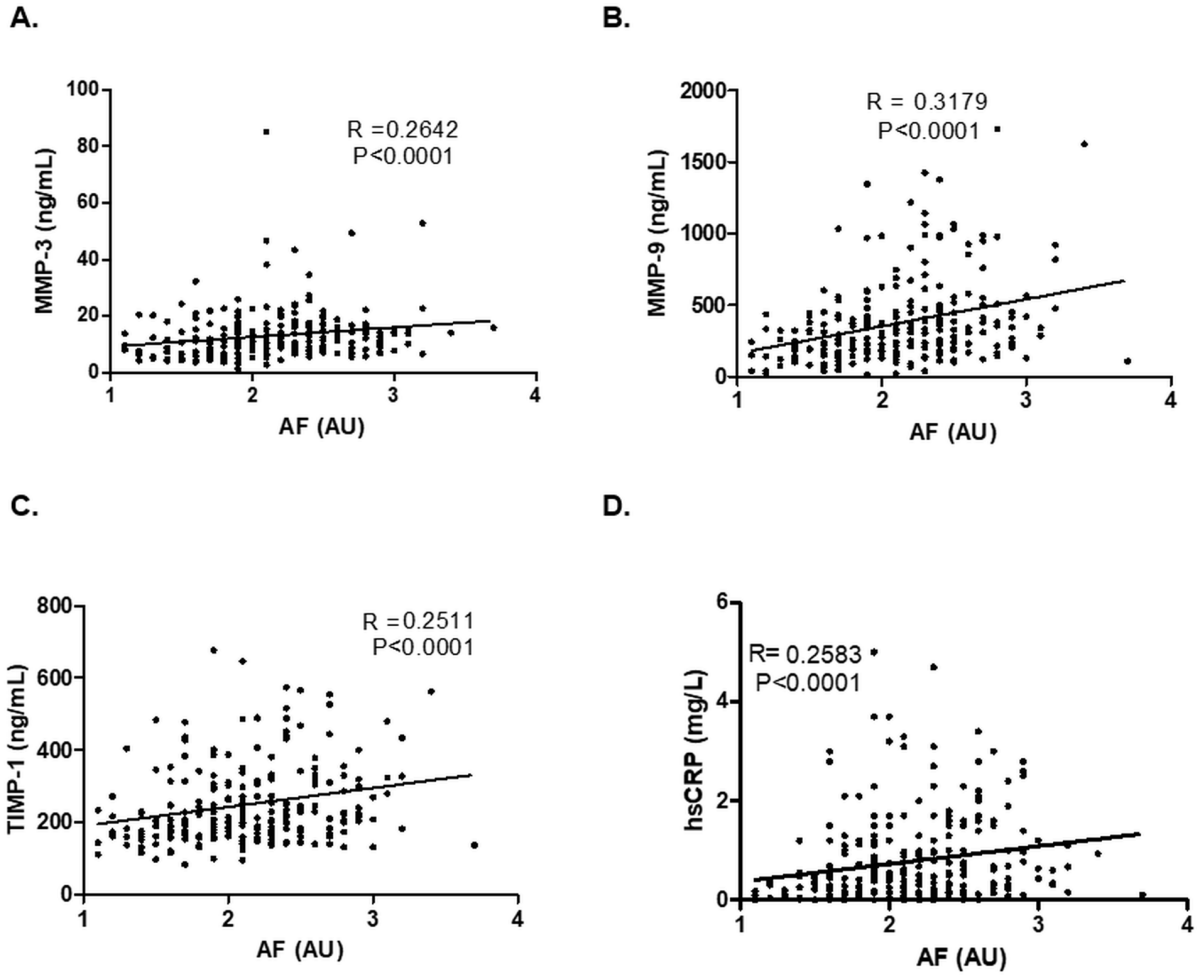
Scatter plots showing the association between skin autofluorescence (AF) and plasma levels of **(A)** MMP-3, **(B)** MMP-9, **(C)** TIMP-1, and **(D)** hsCRP. Lines represent linear regression fits. Correlation coefficients (*r*) and *p*-values are indicated in each panel. AU, arbitrary units; hsCRP, high-sensitivity C-reactive protein; MMP, matrix metalloproteinase; TIMP, tissue inhibitor of metalloproteinases.

In univariable correlation analyses, skin autofluorescence was positively associated with plasma MMP-3 (*r* = 0.26, *p* < 0.0001), MMP-9 (*r* = 0.31, *p* < 0.0001), TIMP-1 (*r* = 0.25, *p* < 0.0001), and hsCRP (*r* = 0.26, *p* < 0.0001) (Figure 1). Univariable linear regression results are presented separately in Table 2. No correlation was found between skin autofluorescence and sRAGE levels (data not shown). MMP-3 and MMP-9 were moderately correlated with TIMP-1 (*r* = 0.37 and *r* = 0.41, respectively; both *p* < 0.001), and weakly with each other (*r* = 0.19, *p* = 0.003; data not shown).

**Table 2.** Univariable and multivariable regression analyses between skin autofluorescence and MMP-3, MMP-9, TIMP-1, and vascular risk factors.

	B (95% CI)	β	*p*
*Univariable*			
MMP-3	0.007 (0.004–0.009)	0.328	< 0.001
MMP-9	0.005 (0.003–0.006)	0.336	< 0.001
TIMP-1	0.009 (0.005–0.012)	0.313	< 0.001
*Multivariable*			
MMP-3^a^	0.004 (0.002–0.006)	0.200	< 0.001
MMP-9^a^	0.002 (0.000–0.004)	0.125	0.050
TIMP-1^a^	0.003 (0.000–0.007)	0.118	0.049
DM (yes/no)	0.002 (0.001–0.004)	0.160	0.003
Age	0.006 (0.004–0.008)	0.355	< 0.001
BMI	< 0.001 (0.000–0.000)	0.290	< 0.001
Smoking (yes/no)	0.001 (0.000–0.003)	0.086	0.107
hsCRP	< 0.001 (–0.002 to 0.001)	–0.052	0.409

Univariable and multivariable regression analyses were performed using a stepwise selection method.

B indicates the unstandardized coefficients with 95% CI, and β indicates standardized coefficients.

MMP-3 and hsCRP were log-transformed; the natural logarithm of age was used.

a MMP-3, MMP-9, and TIMP-1 were analyzed separately as they were closely related.

BMI, body mass index; DM, diabetes mellitus; hsCRP, high-sensitivity C-reactive protein; MMP, matrix metalloproteinase; TIMP, tissue inhibitor of metalloproteinases.

In multivariable linear regression models adjusted for age, BMI, smoking, and diabetes, skin autofluorescence remained independently associated with MMP-3 (*p* < 0.001), MMP-9 (*p* = 0.05), and TIMP-1 (*p* = 0.049), but not with hsCRP (*p* = 0.41) (Table 2).

### Association with carotid intima–media thickness (IMT) and plaque burden

**Figure 2.**
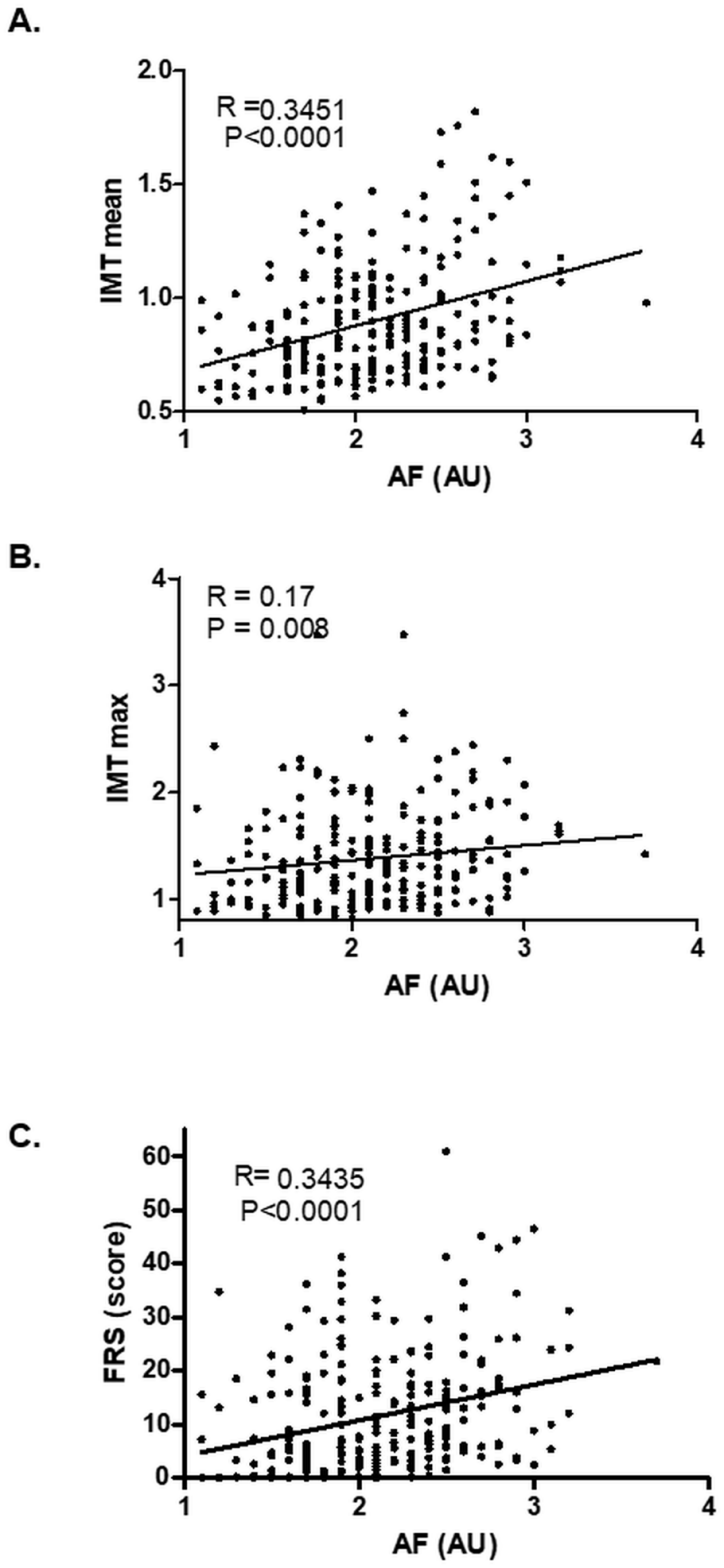
Association between skin autofluorescence (AF) and **(A)** mean carotid intima–media thickness (IMTmean; in mm); **(B)** maximum carotid intima–media thickness (IMTmax; in mm); and **(C)** 10-year cardiovascular risk estimated by the Framingham Risk Score (FRS) (%). Lines represent linear regression fits. AU, arbitrary units.

Skin autofluorescence showed a positive correlation with both IMT_mean_ (*r* = 0.35, *p* < 0.0001) and IMT_max_ (*r* = 0.17, *p* = 0.008) (Figures 2A and 2B). In addition, skin autofluorescence showed a positive association with the FRS, reflecting a higher estimated cardiovascular risk with increasing tissue AGE accumulation (Figure 2C). IMT_mean_ was also associated with MMP-3 (*r* = 0.23, *p* < 0.001), MMP-9 (*r* = 0.31, *p* < 0.001), TIMP-1 (*r* = 0.28, *p* = 0.01), and hsCRP (*r* = 0.16, *p* = 0.01). IMT_max_ correlated with MMP-9 and TIMP-1 (both *r* = 0.15, *p* = 0.02), but not with MMP-3 or hsCRP (data not shown).

**Figure 3.**
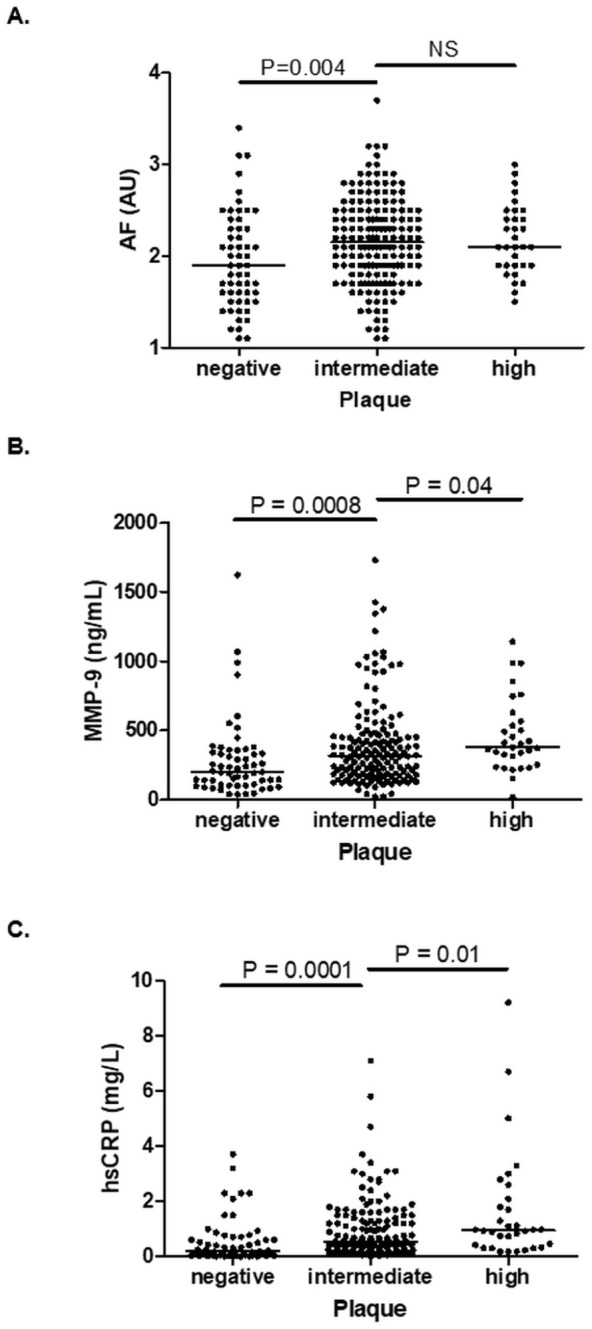
Skin autofluorescence (AF), MMP-9, and hsCRP according to carotid plaque burden. Participants were grouped according to carotid plaque score (negative = 0 segments, intermediate = 1–3 segments, and high = 4+ segments). **(A)** Skin AF was significantly higher in individuals with intermediate plaque burden compared to those without plaques (*p* = 0.004); no significant difference between intermediate and high burden groups. **(B)** Plasma MMP-9 levels were elevated in both intermediate and high plaque burden groups (*p* = 0.0008 and 0.04, respectively). **(C)** hsCRP levels were similarly increased with plaque burden (*p* = 0.0001 and 0.01, respectively). Data are shown as individual values with group medians indicated. AU, arbitrary units; hsCRP, high-sensitivity C-reactive protein; MMP, matrix metalloproteinase; NS, not significant.

Participants were divided into three groups based on carotid plaque score: no plaques (*n* = 64), intermediate burden (one to three segments, *n* = 166), and high burden (four or more segments, *n* = 32). Skin autofluorescence was significantly higher in individuals with intermediate plaque burden compared to those without plaques (*p* = 0.004); however, the difference between the negative plaque group and the high plaque burden group (four or more segments) did not reach statistical significance (Figure 3A).

Plasma MMP-3, MMP-9, TIMP-1, and hsCRP levels were significantly higher in subjects with intermediate plaque burden than in those without plaques (all *p* < 0.001). Furthermore, MMP-9 and hsCRP levels were higher in individuals with a high plaque burden compared to the intermediate group (*p* = 0.04 and *p* = 0.01, respectively) (Figures 3B and 3C). No such differences were observed for MMP-3 or TIMP-1 between the intermediate and high plaque burden groups.

## Discussion

In this study, we demonstrated that skin autofluorescence, a noninvasive marker of tissue AGE accumulation, is independently associated with circulating levels of matrix metalloproteinases (MMP-3 and MMP-9), TIMP-1, and markers of subclinical atherosclerosis in asymptomatic individuals. Among the biomarkers studied, MMP-9 showed the strongest association with both skin autofluorescence and carotid IMT, suggesting a potential mechanistic link between AGE accumulation and matrix remodeling of the arterial wall.

Our findings are consistent with the hypothesis that AGE-related mechanisms contribute to vascular degradation through MMP activation, as suggested by experimental studies of the AGE–RAGE axis. However, as circulating soluble RAGE levels did not differ in our cohort, our data do not provide direct evidence for altered RAGE expression or activation. In vitro and ex vivo studies have shown that AGEs promote MMP expression via RAGE engagement, contributing to inflammation and extracellular matrix breakdown within plaques.^10–12^ AGEs have been found to co-localize with inflammatory cells and regions of plaque vulnerability, supporting their pathological role.^11^

Furthermore, AGE accumulation may contribute to fibrous cap thinning and loss of plaque stability, especially in the presence of DM or oxidative stress. The present data provide clinical, in vivo support for an association between tissue AGE accumulation and markers of vascular matrix remodeling, consistent with mechanisms described in experimental and histopathological studies. Prospective studies incorporating advanced plaque imaging or clinical outcomes are needed to determine whether tissue AGE accumulation is associated with plaque stability or therapeutic response.^24^

The concurrent positive associations of MMPs and TIMP-1 with skin autofluorescence likely reflect coordinated upregulation of matrix remodeling pathways, in which increased TIMP-1 represents a compensatory response to heightened proteolytic activity rather than effective suppression of MMP function.^21^ MMP-9, in particular, has been implicated in plaque rupture and destabilization. Prior studies have shown that MMP-9 is highly expressed in vulnerable carotid and coronary plaques, and that circulating levels increase in acute coronary syndromes.^6,22^ In our study, MMP-9 was not only independently associated with skin autofluorescence and IMT, but also significantly elevated in participants with higher plaque burden. These findings align with earlier reports of elevated MMP-9 activity in unstable lesions.^21^ Additionally, skin autofluorescence was significantly associated with IMT and plaque presence, suggesting that tissue AGE burden reflects early vascular remodeling processes.

Although skin autofluorescence correlated with hsCRP, this association was not independent of traditional risk factors. hsCRP was chosen as a marker of systemic inflammation because it reflects chronic low-grade inflammatory activity and has established relevance in cardiovascular risk assessment, whereas circulating cytokines often show greater biological variability in asymptomatic populations.^17^ This suggests that AGE accumulation is not merely a reflection of systemic inflammation but reflects distinct, chronic metabolic damage to the vascular matrix.

Interestingly, we did not observe a correlation between skin autofluorescence and soluble RAGE (sRAGE). Although previous studies have shown such associations in individuals with coronary artery disease,^22^ it is possible that sRAGE levels fluctuate in response to acute inflammatory events or reflect cleavage rather than expression of full-length RAGE, as previously discussed in the literature.^22^ Additionally, sRAGE measured by ELISA may not differentiate between functionally distinct isoforms. The combined assessment of AGEs, sRAGE, and endogenous secretory RAGE (esRAGE) may therefore offer greater insight.

Skin autofluorescence has been previously linked to cardiovascular morbidity and mortality in patients with diabetes, chronic kidney disease, and peripheral artery disease.^7–9^ Our findings extend this relationship to a subclinical population, and suggest that skin autofluorescence, in combination with MMP profiling, could serve as an early marker for vascular remodeling and risk.

Importantly, skin autofluorescence measurement is operator-independent, rapid, and noninvasive. Unlike serum AGE or MMP quantification, it reflects long-term cumulative metabolic stress in tissue, making it attractive for screening or risk stratification. Given that MMP-9 was the most robustly associated marker, the combination of tissue AGE and circulating MMP-9 may offer a dual marker approach for identifying early plaque activity.

This study has several strengths, including a well-characterized population with a broad spectrum of cardiovascular risk, standardized biomarker and imaging assessments, and comprehensive statistical analyses. However, several limitations should be considered. First, the observed effect sizes and inter-individual variability in skin autofluorescence and circulating MMPs indicate that skin autofluorescence lacks the specificity required for use as a standalone, first-line clinical diagnostic test. Accordingly, the modest correlation coefficients observed limit the predictive utility of skin autofluorescence for identifying individuals with high plaque burden, particularly at the individual level. Rather, skin autofluorescence should be interpreted as a marker of cumulative metabolic stress and early vascular matrix remodeling, reflecting long-term tissue damage rather than acute disease activity. Its potential clinical value therefore lies in adjunctive risk stratification, particularly when combined with complementary biomarkers such as MMP-9 or with vascular imaging markers of subclinical atherosclerosis. Second, the cross-sectional design limits causal inference. Carotid IMT and plaque burden were assessed using two closely related, but not identical, imaging protocols. Although imaging was harmonized, minor variability cannot be excluded. The AGE Reader cannot reliably assess individuals with Fitzpatrick skin types V–VI due to insufficient skin reflectance, limiting generalizability to more diverse populations. Finally, plasma levels of MMPs may not precisely reflect local vascular activity, although histological studies support their biological relevance.^21^ In addition, although TIMP-1 is a key inhibitor of MMP-9 activity, we did not specifically evaluate the MMP-9/TIMP-1 ratio. Circulating levels of MMPs and TIMPs may not directly reflect local proteolytic balance within the arterial wall, and future studies incorporating measures of MMP/TIMP balance at the tissue level may provide additional mechanistic insight.^21^

## Conclusion

Skin autofluorescence is independently associated with circulating levels of MMP-3, MMP-9, and TIMP-1, as well as with IMT and carotid plaque burden. Among these markers, MMP-9 showed the strongest relationship with both skin autofluorescence and subclinical atherosclerosis. These findings provide evidence that AGE accumulation, as measured noninvasively by skin autofluorescence, reflects matrix remodeling and plaque development. The combination of skin autofluorescence and MMP-9 may represent a promising biomarker strategy for early detection of vascular disease. Future prospective studies with imaging and outcome data are needed to validate these markers and their utility in cardiovascular risk stratification.

## References

[1] Libby P Ridker PM Hansson GK. Progress and challenges in translating the biology of atherosclerosis. Nature 2011; 473: 317–325. 21593864 10.1038/nature10146

[2] Goldin A Beckman JA Schmidt AM , et al. Advanced glycation end products: Sparking the development of diabetic vascular injury. Circulation 2006; 114: 597–605. 16894049 10.1161/CIRCULATIONAHA.106.621854

[3] Del Turco S Basta G . An update on advanced glycation endproducts and atherosclerosis. Biofactors 2012; 38: 266–274. 22488968 10.1002/biof.1018

[4] Bierhaus A Humpert PM Morcos M , et al. Understanding RAGE, the receptor for advanced glycation end products. J Mol Med 2005; 83: 876–886. 16133426 10.1007/s00109-005-0688-7

[5] Back M Ketelhuth DF Agewall S. Matrix metalloproteinases in atherothrombosis. Prog Cardiovasc Dis 2010; 52: 410–428. 20226959 10.1016/j.pcad.2009.12.002

[6] Loftus IM Naylor AR Goodall S , et al. Increased matrix metalloproteinase-9 activity in unstable carotid plaques: A potential role in acute plaque disruption. Stroke 2000; 31: 40–47. 10625713 10.1161/01.str.31.1.40

[7] de Vos LC Mulder DJ Smit AJ , et al. Skin autofluorescence is associated with 5-year mortality and cardiovascular events in patients with peripheral artery disease. Arterioscler Thromb Vasc Biol 2014; 34: 933–938. 24526694 10.1161/ATVBAHA.113.302731

[8] Noordzij MJ Lefrandt JD Loeffen EA , et al. Skin autofluorescence is increased in patients with carotid artery stenosis and peripheral artery disease. Int J Cardiovasc Imaging 2012; 28: 431–438. 21336554 10.1007/s10554-011-9805-6 PMC3288376

[9] Mulder DJ van Haelst PL Graaff R , et al. Skin autofluorescence is elevated in patients with stable coronary artery disease and is associated with serum levels of neopterin and the soluble receptor for advanced glycation end products. Atherosclerosis 2008; 197: 217–223. 17499742 10.1016/j.atherosclerosis.2007.03.027

[10] Rittie L Berton A Monboisse JC , et al. Decreased contraction of glycated collagen lattices coincides with impaired matrix metalloproteinase production. Biochem Biophys Res Commun 1999; 264: 488–492. 10529390 10.1006/bbrc.1999.1519

[11] Hanssen NM Wouters K Huijberts MS , et al. Higher levels of advanced glycation endproducts in human carotid atherosclerotic plaques are associated with a rupture-prone phenotype. Eur Heart J 2014; 35: 1137–1146. 24126878 10.1093/eurheartj/eht402

[12] Zhang F Banker G Liu X , et al. The novel function of advanced glycation end products in regulation of MMP-9 production. J Surg Res 2011; 171: 871–876. 20638679 10.1016/j.jss.2010.04.027 PMC3623272

[13] Meerwaldt R Graaff R Oomen PH , et al. Simple non-invasive assessment of advanced glycation endproduct accumulation. Diabetologia 2004; 47: 1324–1330. 15243705 10.1007/s00125-004-1451-2

[14] Baldassarre D Nyyssönen K Rauramaa R , et al. Cross-sectional analysis of baseline data to identify the major determinants of carotid intima-media thickness in a European population: The IMPROVE study. Eur Heart J 2010; 31: 614–622. 19952003 10.1093/eurheartj/ehp496

[15] Fitzpatrick TB. The validity and practicality of sun-reactive skin types I through VI. Arch Dermatol 1988; 124: 869–871. 3377516 10.1001/archderm.124.6.869

[16] von Elm E Altman DG Egger M , et al. The STROBE statement: Guidelines for reporting observational studies. Lancet 2007; 370: 1453–1457. 18064739 10.1016/S0140-6736(07)61602-X

[17] Wilson PW D’Agostino RB Levy D , et al. Prediction of coronary heart disease using risk factor categories. Circulation 1998; 97: 1837–1847. 9603539 10.1161/01.cir.97.18.1837

[18] Smit AJ Gerrits EG. Skin autofluorescence as a measure of advanced glycation endproduct deposition: A novel risk marker in chronic kidney disease. Curr Opin Nephrol Hypertens 2010; 19: 527–533. 20844429 10.1097/MNH.0b013e32833e9259

[19] Mulder DJ Water TV Lutgers HL , et al. Skin autofluorescence, a novel marker for glycemic and oxidative stress-derived advanced glycation endproducts: An overview of current clinical studies, evidence, and limitations. Diabetes Technol Ther 2006; 8: 523–535. 17037967 10.1089/dia.2006.8.523

[20] Taurino M Raffa S Mastroddi M , et al. Metalloproteinase expression in carotid plaque and its correlation with plasma levels before and after carotid endarterectomy. Vasc Endovascular Surg 2007; 41: 516–521. 18166633 10.1177/1538574407307405

[21] Choudhary S Higgins CL Chen IY , et al. Quantitation and localization of matrix metalloproteinases and their inhibitors in human carotid endarterectomy tissues. Arterioscler Thromb Vasc Biol 2006; 26: 2351–2358. 16888239 10.1161/01.ATV.0000239461.87113.0b

[22] Park HJ Baek JY Shin WS , et al. Soluble receptor of advanced glycated endproducts is associated with plaque vulnerability in patients with acute myocardial infarction. Circ J 2011; 75: 1685–1690. 21576827 10.1253/circj.cj-10-1248

[23] Touboul PJ Hennerici MG Meairs S , et al. Mannheim carotid intima-media thickness and plaque consensus (2004–2006–2011). Cerebrovasc Dis 2012; 34: 290–296. 23128470 10.1159/000343145 PMC3760791

[24] Puri R Worthley MI Nicholls SJ. Intravascular imaging of vulnerable coronary plaque: Current and future concepts. Nat Rev Cardiol 2011; 8: 131–139. 21263456 10.1038/nrcardio.2010.210

